# Case Report: Pathogenic *PNPLA2* variants and nonsense-mediated mRNA decay result in an early-onset neutral lipid storage disease with myopathy

**DOI:** 10.3389/fgene.2025.1642442

**Published:** 2025-08-21

**Authors:** Sara Missaglia, Eleonora Martegani, Corrado Angelini, Rita Horvath, Veronika Karcagi, Endre Pal, Daniela Tavian

**Affiliations:** ^1^ Laboratory of Cellular Biochemistry and Molecular Biology, CRIBENS, Catholic University of the Sacred Heart, Milan, Italy; ^2^ Department of Psychology, Catholic University of Sacred Heart, Milan, Italy; ^3^ Campus Pietro d’Abano, Department of Neurosciences, University of Padua, Padua, Italy; ^4^ Department of Clinical Neurosciences, University of Cambridge, Cambridge, United Kingdom; ^5^ Istenhegyi Genetic Diagnostic Centre, Molecular Genetic Laboratory, Budapest, Hungary; ^6^ Department of Neurology, Medical School, University of Pécs, Pécs, Hungary; ^7^ Department of Pathology, Neuropathology Unit, Medical School, University of Pécs, Pécs, Hungary

**Keywords:** case report, early onset myopathy, NLSDM, ATGL, nonsense-mediated RNA decay, no protein production

## Abstract

Neutral Lipid Storage Disease with Myopathy (NLSDM) is a rare lipid metabolism disorder caused by impaired Adipose Triglyceride Lipase (ATGL) activity, leading to neutral lipid accumulation in various tissues. It typically manifests with progressive skeletal myopathy, with an onset of around 35 years. In addition, some patients develop cardiomyopathy and liver dysfunction. Herein, we report the molecular characterization of a 27-year-old Hungarian patient and his family in whom two severe *PNPLA2* mutations led to complete loss of ATGL production in the patient’s tissues. DNA sequencing revealed a nonsense (c.24G>A) and a frameshift mutation (c.798dupC) in the *PNPLA2* gene. RNA analysis showed nonsense-mediated decay of the c.798dupC transcript, while c.24G>A was normally expressed in the patient. However, Western blot analysis did not detect ATGL protein production. From a clinical perspective, our patient exhibited pes planus, proximal muscle weakness of the lower limbs and elevated CK levels from the age of six. MRI and biopsy revealed lipid accumulation, and leukocytes showed Jordans’ anomaly. The muscle weakness progressed, and cardiomyopathy and hepatic steatosis were also observed recently. The case highlights two severe *PNPLA2* mutations leading to complete ATGL deficiency and an unusual early-onset myopathy in childhood.

## 1 Introduction

Adipose Triglyceride Lipase (ATGL) is a key enzyme involved in lipid metabolism in human cells. Indeed, ATGL catalyzes the first step of triacylglycerols (TAGs) breakdown stored into lipid droplets (LDs), the main depots of neutral lipids in cells. TAGs are hydrolyzed into diacylglycerols (DAGs) and free fatty acids (FFAs) that are necessary during energy requirement when lipolysis pathway is activated. ATGL is highly expressed in white and brown adipocytes, but is also expressed in other tissues including heart, skeletal muscle, liver, pancreas, lung, retina, and immune cells (Schweiger et al., 2009; Schreiber et al., 2019). ATGL is encoded by *patatin-like phospholipase domain containing 2* (*PNPLA2*) gene and is composed of 504 amino acids with a patatin domain (amino acids from 10 to 178) containing the active site residues (S47 and D166) at the N-terminal region. A hydrophobic LD binding site is localized in the C-terminal part (amino acids from 315 to 360) (Missaglia et al., 2019).

Mutations in *PNPLA2* gene cause the development of a rare autosomal recessive muscle disorder known as Neutral Lipid Storage Disease with Myopathy (NLSDM, OMIM #610717) (Fischer et al., 2007). Indeed, changes in normal expression or function of ATGL lead to an abnormal accumulation of TAGs in multiple tissues and organs, especially in skeletal muscle and heart (Pasanisi et al., 2016; Missaglia et al., 2017).

Manifestation of NLSDM usually begins with proximal and distal muscle weakness of upper and lower limbs and elevated levels of serum creatine kinase (CK) (Laforêt et al., 2013; Kaneko et al., 2014). Then, all patients develop progressive skeletal muscle myopathy and almost half of them also present cardiomyopathy. Indeed, ATGL plays an important role in myocardial cytosolic lipolysis, and defects in FFAs metabolism can compromise proper heart functioning (Wang et al., 2024; Fonseka et al., 2025). In addition, symptoms can include hepatomegaly, diabetes mellitus, chronic pancreatitis, and sensorineural hearing loss (Kaneko et al., 2014). Moreover, histological analyses of patients’ blood reveal Jordans’ anomaly in leukocytes [3].

More than 70 *PNPLA2* mutations have been described until now in almost 130 patients worldwide (Missaglia et al., 2022; Wang et al., 2024). NLSDM is a highly variable disease ranging from mild symptoms to more severe ones. Clinical phenotypes may depend on the type of mutations in *PNPLA2* gene causing total or partial loss of enzyme activity. However, since the same mutation can lead to different clinical manifestation, a genotype-phenotype correlation is not clear yet (Pennisi et al., 2017; Zhang et al., 2019).

In this study, we reported the clinical and molecular characterization of the first Hungarian patient and his family members, in which two different mutations in *PNPLA2* gene were identified.

## 2 Methods

### 2.1 Clinical evaluation

Muscle histopathology was assessed following the standard methodology outlined by Dubowitz. Haematoxylin and eosin (H&E) and Oil Red O (ORO) staining were performed on frozen cross-sections of muscle biopsies.

Peripheral blood samples treated with EDTA were collected and centrifuged at 3,300 × g for 10 min. The resulting buffy coats were smeared onto microscope slides, air-dried thoroughly, and fixed. Staining was performed using the May-Grünwald–Giemsa (MGG) and Oil Red O, following established protocols.

### 2.2 Molecular analysis

Peripheral blood mononuclear cells (PBMCs) were isolated by density gradient centrifugation using Histopaque, following the manufacturer’s instructions. Genomic DNA was obtained using a Puregene DNA Isolation kit (Qiagen, Venlo, Netherlands), while total RNA was extracted with Trizol solution and reverse-transcribed as reported previously (Tavian et al., 2021). *PNPLA2* exons were amplified and PCR products purified by NucleoSpin Gel and PCR Clean-up kit (Macherey-Nagel, Germany), as previously reported (Tavian et al., 2021).

ATGL protein expression was assessed using PBMCs fraction after protein extraction (RIPA buffer–ThermoFisher Scientific) and quantification (BCA protein assay–ThermoFisher Scientific), following manufacturer’s instructions. Anti-ATGL Polyclonal Antibody was purchased by Invitrogen (catalog #PA5-17436).

## 3 Case description

**FIGURE 1:**
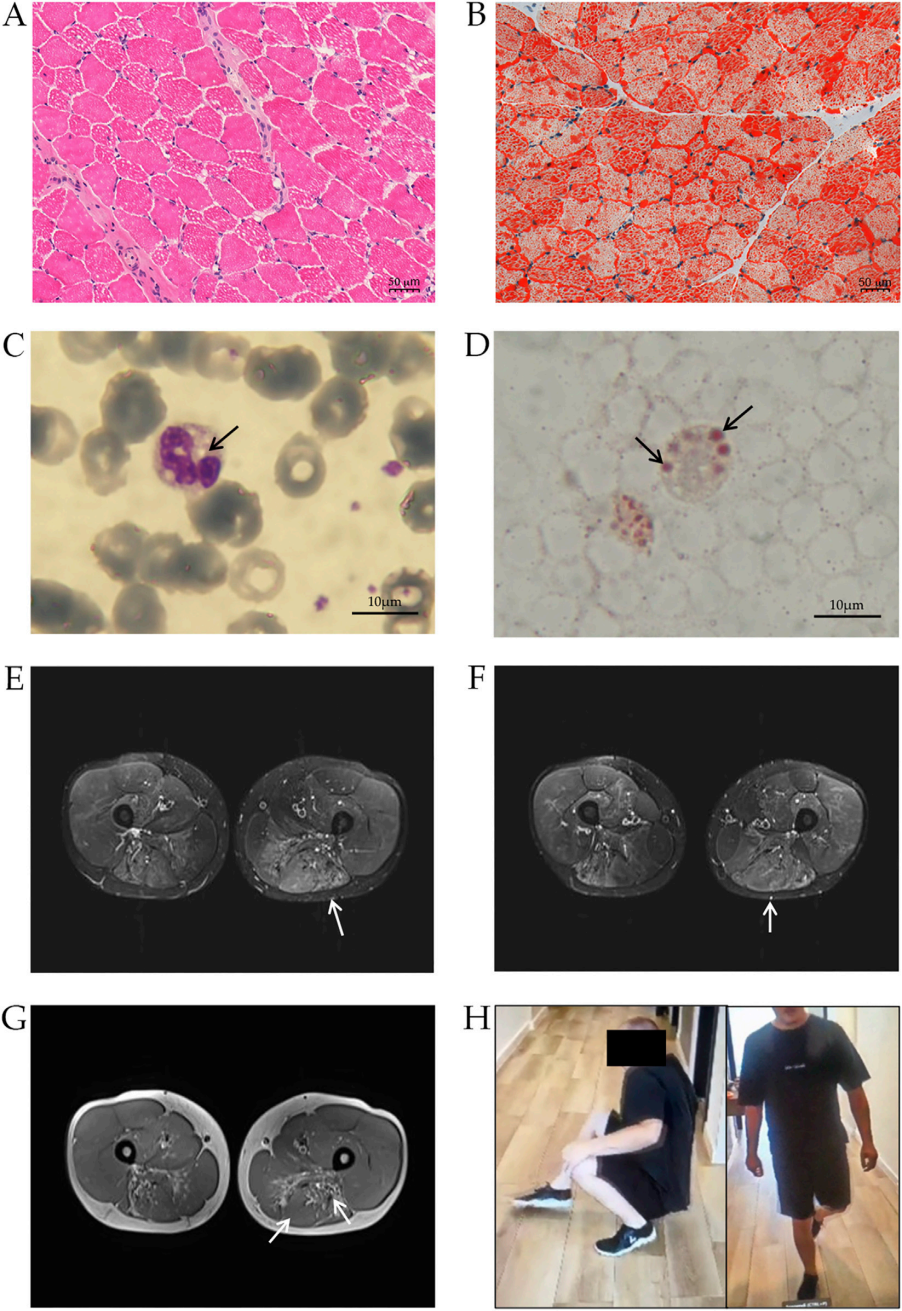
Clinical characterization of NLSDM patient. Muscle histopathology of patient’s biopsy, stained with haematoxylin-eosin **(A)** and Oil Red-O **(B)** Peripheral blood smear stained with May-Grünwald-Giemsa **(C)** and Oil Red-O **(D)** shows the presence of LDs (arrows) in leukocytes. **(E)** T2-weighted STIR. Pronounced oedema can be noted in the affected semitendinosus muscles (arrow). Currently, no significant atrophy can be detected in the quadriceps femoris muscles, however, diffuse, mild, symmetrical oedema is also noted in these muscles, predominantly in the distal portions. Subtle oedema can be noted in the adductors (arrow) and semitendinosus (arrow) **(F)**. **(G)**. MRI scan of the soft tissue of the thigh. Moderate atrophy can be identified in the flexor muscle groups symmetrically bilaterally, mainly in the distal portions of the biceps femoris muscles (arrow) and the semimembranosus muscles (arrow). **(H)** Patient’s photographs during Gowers’ maneuver (left) and during stepping gait (right).

The patient is a Hungarian male of 27 years old, born to non-consanguineous parents (Supplementary Figure S1). Clinical manifestations began at age six when, during an orthopedic visit, the patient manifested with some foot weakness and pes planus. He had elevated CK levels (1350 IU/L), muscle biopsy revealed lipid accumulation, and he was positive for Jordans’ anomaly test (Figures 1A,D).

At age 22, the patient suffered from a distal and proximal weakness of the lower limbs, with minimal weakness to upper limb and shoulder girdle. Magnetic Resonance Imaging (MRI) revealed diffuse oedema of thigh muscles (Figures 1E,F) and atrophy to thigh flexors muscles (biceps femoris, semimembranosus muscles) (Figure 1G), indicating a progression of the disease. Diffuse abnormalities were also observed in the legs, particularly in the anterior muscle compartment.

Patient’s upper limb musculature was normotrophic with moderate scapular winging (scapula alata). Manual muscle testing (MRC scale) showed muscle weakness in shoulder muscles 4+/5, arm rotator muscles 4+/5, without significant weakness in other muscle groups of the upper limbs. Hip muscles strength was retained. In the lower limbs, thigh muscles were slightly hypotrophic, while calf and lower leg muscles were markedly hypotrophic. Foot dorsiflexion was 2/5, and plantarflexion was 4-/5 (MRC scale). He has difficulty standing on tiptoe and could not stand on his heel. He could stand up easily from squatting, however he had difficulty during Gowers’ maneuver. There was areflexia in all limbs, and his gait was stepping (Figure 1H). Coordination and stance were normal, speech was intact, and he was alert and oriented. A cardiac MRI revealed hypertrophic cardiomyopathy (HCM) with inferoseptal hypertrophy of 15 mm. Recently, transient elastography has revealed hepatic steatosis.

The father is a 59-years-old overweight man, with lower physical fitness compared to his peers. He reported having less physical strength and exertional capacity at a young age, for example, he was unable to carry as much weight or cycle as long as other schoolmates. Currently, he does not experience difficulties in daily life, however his level of physical activity is quite low.

The mother, 52 years old, recently suffered from weakness of her upper limbs and had difficulty swallowing. The sister of the patient is a healthy woman of 30 years without clinical implications.

## 4 Molecular and genetic characterization

**FIGURE 2:**
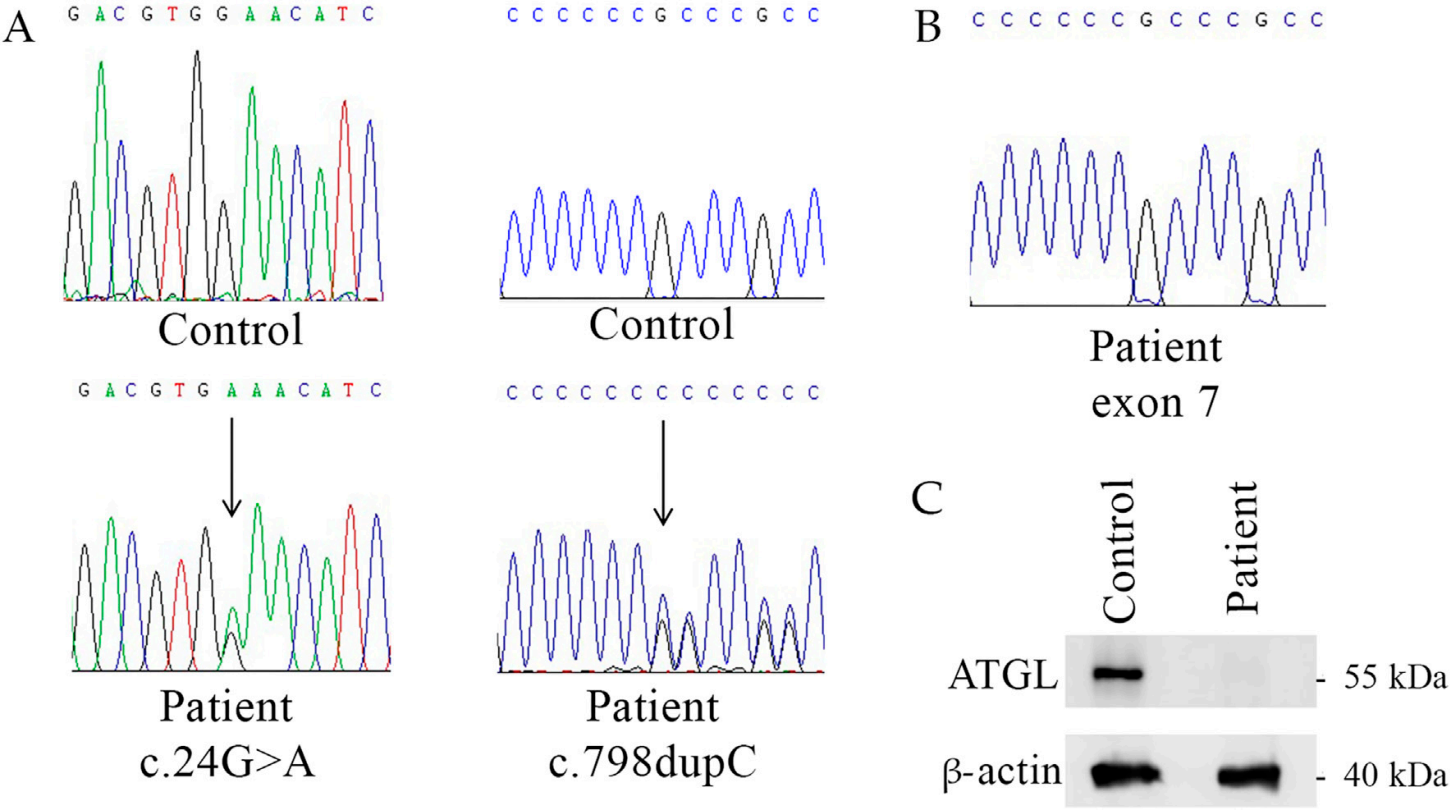
Genetic and molecular characterization of NLSDM patient. **(A)** Patient’s genomic DNA electropherograms of *PNPLA2* exon 2 and exon 7. **(B)** cDNA electropherogram of exon 7 of NLSDM patient does not show the **(C)**. 798dupC allele. **(C)** Western blot analyses for the expression of ATGL in the patient and his sister (healthy control).

Diagnosis of NLSDM derived from genetic analysis of peripheral blood (Baldo et al., 2016). *PNPLA2* exon sequencing revealed that the patient is a compound heterozygote carrying the nonsense mutation c.24G>A (p.W8*) in exon 2 and the frameshift mutation c.798dupC in exon 7 (Figure 2A). Sequencing analyses also revealed that the patient inherited from his mother the c.24G>A mutation, while the father carried the c.798dupC allele. The sister of the patient was a healthy control without mutations in *PNPLA2* gene.

To determine the pathogenetic impact of DNA mutations on ATGL production we analyzed the RNA extracted from patient’s blood sample. Sequencing of *PNPLA2* exons from the patient revealed the c.24G>A mutation in exon 2, while c.798dupC allele was not detected (Figure 2B). This result suggests the absence of the mRNA carrying the frameshift mutation.

Western blot analyses revealed the absence of ATGL in patient’s sample, compared to his sister, the healthy control, as shown in Figure 2C.

## 5 Discussion

We presented the first NLSDM Hungarian family with a male patient characterized by an early onset at age 6. The average age of NLSDM onset is usually around the third/fourth decade (Zhang et al., 2019; Fu et al., 2023). However, a small minority of patients have been described manifesting symptoms during infancy or childhood (0–10 years) (Zhang et al., 2019; Fu et al., 2023). Early signs of NLSDM mainly appear as exercise intolerance, asymptomatic hyperCKemia, and rarely mild hypotonia and myopathy. In our patient pes planus and some mild foot muscle weakness in addition to elevated CK serum levels were observed originally. Current pathological conditions include persistent and progressive muscle weakness, more pronounced in distal lower extremities, and muscle atrophy in proximal lower limbs characterized by symmetrical progression. Hypertrophic cardiomyopathy and hepatic steatosis were recently observed. Cardiomyopathy was reported in half of 130 NLSDM cases (47%) and is mainly diagnosed after the third decade of age, indicating a late involvement of the heart. Liver involvement was found in less than 15% of the total of the cases and usually is presented as hepatomegaly, while rarely (5%) patients manifested fatty liver (Missaglia et al., 2019; Shi et al., 2021; Yamada et al., 2023).

Clinical symptoms of NLSDM are associated with abnormal accumulation of lipids in patients’ tissues due to the reduced or absent activity of ATGL lipase. The severity of manifestations is usually linked to the impairment in ATGL activity and, in general, mutations that lead to dramatic decrease in lipase production may undergo more serious clinical manifestation (Tavian et al., 2021). However, the relationship between genotype and phenotypic manifestation, including the age of onset, remains unclear.

Here we report a patient carrying a novel combination of mutated *PNPLA2* alleles: the c.24G>A (p.W8*) variation, inherited from the mother, and the c.798dupC mutation of paternal origin, both showing the total loss of ATGL protein. The mutant transcript of c.24G>A allele should encode an eight-amino acid-long peptide (p.W8*), however it is likely that it has no functional relevance. The same mutation was previously identified in two Italian patients (Tavian et al., 2012). These two siblings were compound heterozygotes for the c.24G>A mutation and the c.516C>A (p.N172K) missense mutation. Previous functional studies showed that ATGL (N172K) preserved a partial lipase activity in patients’ tissues (Tavian et al., 2012). In these subjects clinical manifestations began late, at ages 25 and 40, with leg myalgia and cramps, respectively.

The frameshift mutation c.798dupC was previously identified by Jousserand and colleagues in a male patient who was homozygote for this *PNPLA2* variation. He suffered from distal skeletal myopathy and severe cardiomyopathy, and his diagnosis was confirmed at age 54 (Jousserand et al., 2016). Nevertheless, the patient reported difficulties performing physical activities as early as his school years.

The c.798dupC mutation is predicted to produce an ATGL truncated protein of 266 wild type amino acids and further 39 residues (p.A267Rfs*40). Nevertheless, our molecular analyses highlighted the absence of the c.798dupC mutated transcript and of the ATGL truncated protein. The c.798dupC mutation causes a shift in the reading frame that alters the amino acid sequence starting from codon 267 and leads to a premature termination codon (PTC) at the end of exon 7. Given that the canonical *PNPLA2* transcript comprises 10 exons, this PTC lies well upstream of several exon–exon junctions (exons 8–10) and thus fulfils the structural prerequisites for nonsense-mediated mRNA decay (NMD) activation (Lejeune, 2022; Miller and Pearce, 2014; Carrard and Lejeune, 2023). In the case of *PNPLA2*, the PTC generated by c.798dupC is expected to leave downstream intact EJCs, which can recruit the NMD machinery during the pioneer round of translation, leading to rapid degradation of the mutant transcript. This mechanism is particularly stringent when the PTC occurs in middle exons, such as exon 7 in *PNPLA2*. As described by Lejeune (Lejeune, 2022), these exons are typically flanked by multiple splice junctions that ensure EJC-dependent NMD activation, thereby preventing any stable accumulation of the mutant mRNA. This may explain the absence of detectable ATGL protein in the patient, since the transcript is likely degraded before any significant translation occurs.

Moreover, NMD efficiency varies significantly depending on cellular context, developmental stage, and type of tissue (Carrard and Lejeune, 2023). This variability is influenced by differential expression of core NMD factors, including UPF1, UPF3X, SMG6, and auxiliary modulators such as RNPS1 and PNRC2. The interaction of these factors defines the functional capacity of NMD in each cell type. This may be particularly relevant in NLSDM, where skeletal muscle is the principal tissue affected by LD accumulation due to defective ATGL activity.

Until now, more than 70 different *PNPLA2* mutations have been reported, and 24 of them are frameshift mutations, predicted to produce a mutated ATGL protein. In these cases, the mechanism of NMD should be considered, since it may play an important role in degrading aberrant transcripts and dramatically reduce ATGL production (Samukawa et al., 2020).

The mother of our patient is heterozygote for the *PNPLA2* gene, carrying c.24G>A allele. She recently manifested light signs of muscle weakness, underlining the pathogenic load of this variant. Exercise intolerance in youth was also manifested by the father of our patient, who has a wild-type allele and the c.798dupC mutation. However, we cannot exclude that symptoms manifested by parents could be associated with ageing rather than to the storage disease.

In summary, we reported a novel combination of two pathogenetic *PNPLA2* variants in a male patient, resulting in a complete loss of ATGL production in the patient’s tissues and an early onset of the disease with progressive muscle weakness. NLSDM symptoms are heterogeneous, and the same genetic mutations may not always lead to identical clinical manifestations. This variability could be related to other factors, such as modifier genes, living environments, and patients’ lifestyles. The involvement of mRNA decay in the pathogenicity of NLSDM should also be taken into consideration since it could impair the production of ATGL lipase and worsen patients’ clinical conditions.

## Data Availability

The data that support the findings of this study are available from Daniela Tavian, Catholic University of the Sacred Heart, Milan (Italy).
