# Supplementary material for: Case Report: Pathogenic PNPLA2 variants and nonsense-mediated mRNA decay result in an early-onset neutral lipid storage disease with myopathy
Source: Front Genet. 2025 Aug 21;16:1642442. doi: 10.3389/fgene.2025.1642442 (PMC12408259; doi:10.3389/fgene.2025.1642442)
Supplement: Supplementary file 1 [file Table1.docx]

Supplementary Material


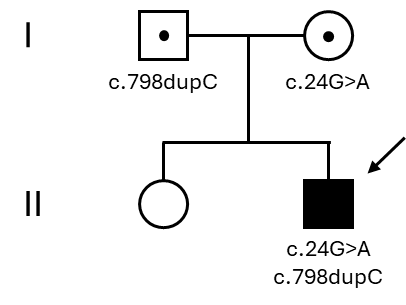


**Supplementary Figure 1.** Pedigree of the family with mutations in *PNPLA2* gene. Arrow indicates the proband.
